# TGFβs Modulate Permeability of the Blood-Epididymis Barrier in an *In Vitro* Model

**DOI:** 10.1371/journal.pone.0080611

**Published:** 2013-11-13

**Authors:** Angelika Stammler, Dieter Müller, Yoshiaki Tabuchi, Lutz Konrad, Ralf Middendorff

**Affiliations:** 1 Institute of Anatomy and Cell Biology, Justus-Liebig-University Giessen, Giessen, Germany; 2 Department of Gynecology and Obstetrics, Justus-Liebig-University Giessen, Giessen, Germany; 3 Division of Molecular Genetics Research, Life Science Research Center, Graduate School of Medicine Pharmaceutical Sciences, University of Toyama, Toyama, Japan; Emory University School of Medicine, United States of America

## Abstract

The blood-epididymis barrier (BEB) is formed by epithelial tight junctions mediating selective permeability of the epididymal epithelium. Defective barrier function can disturb the balance of the epididymal milieu, which may result in infertility. The stroma of the epididymis contains high amounts of cytokines of the TGFβ family of unknown function. We screened possible effects of all three TGFβ isoforms on paracellular tightness in a BEB *in vitro* model based on the strongly polarized mouse epididymal epithelial MEPC5 cells in the transwell system. In this model we found a robust transepithelial electrical resistance (TER) of about 840 Ω x cm^2^. Effects on the paracellular permeability were evaluated by two methods, TER and FITC-Dextran-based tracer diffusion assays. Both assays add up to corresponding results indicating a time-dependent disturbance of the BEB differentially for the three TGFβ isoforms (TGFβ3>TGFβ1>TGFβ2) in a TGFβ-recetor-1 kinase- and Smad-dependent manner. The tight junction protein claudin-1 was found to be reduced by the treatment with TGFβs, whereas occludin was not influenced. Epididymal epithelial cells are predominantly responsive to TGFβs from the basolateral side, suggesting that TGFβ may have an impact on the epididymal epithelium from the stroma *in vivo*. Our data show for the first time that TGFβs decrease paracellular tightness in epididymal epithelial cells, thus establishing a novel mechanism of regulation of BEB permeability, which is elementary for sperm maturation and male fertility.

## Introduction

The epididymis mediates maturation, transport and protection of sperm in the luminal fluid [1]. The epididymal duct is a continuous tube of a length of 1 m in mouse, 3 m in rat and 6 m in human [2]. The duct is formed by a pseudostratified columnar epithelium surrounded by a thin layer of muscle cells [1].

The blood-epididymis barrier (BEB) protects sperm during storage and maturation and controls the state of luminal fluid [1]. The barrier is composed of tight junctions between the epithelial cells. In epididymis, tight junction proteins claudin-1, -3, -4, -7 and occludin were found to be expressed [3], [4]. In *in vitro* cell culture models, the knockdown of one of these claudins (1, -3, -4, or -7) resulted in dramatically decreased transepithelial electrical resistance (TER) tested in human epididymal cell lines [5]. Claudin-1 knockout mice die immediately after birth due to dehydration caused by lack of epidermal barrier. Thus, claudin-1 seems to be pivotal for survival and cannot be compensated by other tight junction proteins [6]. The role of occludin for barrier formation is controversial [7] since occludin knockout mice are viable and have an intact barrier in the intestine and bladder [8]. However, male occludin knockout mice are infertile [9].

Quantitative changes of tight junction proteins on the cell surface have been reported to be caused by diverse substances or processes. The decrease of tight junctions on the cell surface connected with disruption of epithelial barrier has been reported for toxins such as cadmium chloride in the seminiferous epithelium [10] but also for diverse cytokines associated with inflammation and immunoregulation, such as TNFα, IFN-γ, interleukins [11], [12], [13], [14] or TGFβ. TGFβ was reported to increase permeability in diverse epithelia, such as trachea epithelium [15], the seminiferous epithelium [16] and ovarian surface epithelium [17] as well as between the cells of the blood-brain barrier [18]. Several TGFβ pathways have been reported to mediate quantitative changes of tight junctions. In the murine trachea, the JNK pathway was described to be essential for TGFβ response [15], whereas in the seminiferous epithelium Ras/ERK pathway was reported to mediate the signal [16]. In the blood-brain barrier, changes in the permeability were found to be mediated by Smad signaling [18].

TGFβs are important regulators of growth and development and play a pivotal role in immunoregulation. In mammals three isoforms of TGFβs have been identified, TGFβ1, TGFβ2 and TGFβ3. Although the isoforms are mostly described as functionally overlapping, isoform-specific knockout mice revealed non-redundant phenotypes. TGFβ1 knockout mice typically die prenatally due to yolk sac defects; the survivors developed inflammatory disorders and eventually died within a few weeks after birth [19], [20], [21]. TGFβ2 knockout mice have defects in different organs such as heart, kidney, testis as well as various craniofacial defects, axial and appendicular skeletal defects and die perinatally [22]. TGFβ3 knockout mice also die perinatally due to developmental defects of the lung [23]. TGFβ3 knockout mice also show defective palatogenesis [24].

TGFβs are secreted as non-covalent complexes associated with the latency-associated peptides (LAPs). After activation TGFβs bind to TGFβ-receptor-2 (TGFβ-R2), which in turn dimerizes with TGFβ-R1. In response to the binding of the ligand, the intracellular kinase domain of TGFβ-R1 phosphorylates Smad2 or Smad3 that eventually act as transcription factors together with Smad4 [25].

In the epididymis, high amounts of TGFβs were found [26], [27], [28]. TGFβ1 mRNA was described to be present in the stroma of all regions of rat epididymis analyzed by Northern blot [26]. In the same study, TGFβ3 mRNA was predominantly found in the corpus region of epididymis, whereas TGFβ2 mRNA was not detected [26].

In our investigation we aimed to test the influence of cytokines of the TGFβ family on the barrier of epididymal epithelium. For this purpose we used an *in vitro* model of blood-epididymis barrier based on polarized mouse epididymal cells of the cell line MEPC5 [29] cultured on transwell inserts. Cell line MEPC5 was previously established by us and characterized in detail [29]. In this model we evaluated paracellular permeability by two methods, the measurement of transepithelial electrical resistance (TER) and by tracer diffusion. We observed that TGFβs increase paracellular permeability significantly within 4 h depending on (i) the isoform of TGFβ, (ii) the side of application (apical vs. basolateral), and (iii) TGFβ-R1 kinase activity. Tight junction protein claudin-1 was found to be reduced by the treatment with TGFβs (whereas occludin was not influenced), suggesting a TGFβ-R1 kinase-dependent effect on distinct tight junction proteins modulating the BEB.

## Methods

### Cell culture and viability assay

MEPC5 cell line [29] was cultured in DMEM supplemented with 10% FCS and penicillin/streptavidin at 33°C/5% CO_2_/96% humidity. After 2–3 days of culture, cells were detached from 75 cm^2^ flasks using accutase (PAA, Cölbe, Germany) and were seeded (3×10^5^ cells/cm^2^) in 12-well plates. After 24 h of starvation with 2% FCS, TGFβ-1, -2 or -3 human recombinant protein (Promokine, Heidelberg, Germany) were added combined with fresh medium in a final concentration of 10 ng/ml, as described previously for other cell lines [30]. After 24 h of TGFβ treatment, cells were detached and analyzed concerning viability and number of cells using BioRad Cell Counter System (BioRad, Hercules, CA, USA) combined with Trypan blue (BioRad) staining.

### Measurement of transepithelial electrical resistance (TER)

For TER measurement, cells were seeded (3×10^5^ cells/cm^2^) on ThinCert inserts (Greiner, Frickenhausen, Germany) with 0.4 µm pore size in 24-well plates (0.33 cm^2^ per insert) in culture conditions described above. TER measurements were conducted using Millicell ERS-2 (Merck Millipore, Billerica, MA, USA). TER was calculated according to the instructions of the Millicell ERS-2 manual, subtracting the background resistance of blank filters in the same experiment and multiplying the TER [Ω] with the area of the monolayer [cm^2^].

Under these conditions, the TER increases from about 200 Ω x cm^2^ within one day after seeding to about 840 Ω x cm^2^ on second day after seeding, correlating with increasing cell number. After day 2 no further increase of cell number could be observed. From this point on further increase of TER slowed down. To standardize experimental conditions, all experiments were performed between day 2 and day 3 despite of ongoing slight increase of TER especially at the end of day 3 (“24 h” i.e. 72 h after seeding). In order to allow a more convenient comparison of experiments, values were normalized to the initial value at 0 h (i.e. 48 h after seeding) for each individual insert. This 0 h value was described as 100%.

Between day 1 and day 2 cells were starved for 24 h with 2% FCS. At day 2, after measurement of the initial value (0 h), TGFβ-1, -2 or -3 (Promokine) were added in a final concentration of 10 ng/ml combined with fresh medium. For inhibitor experiments, the specific inhibitors were added 3 h prior to TGFβ addition. After the first 6 h medium supplemented with the specific stimulants was exchanged. Inhibitor Ly364947 (Sigma-Aldrich, St Louis, MO, USA) was dissolved 5 mg/ml in DMSO (Sigma-Aldrich) and stored at −20°C. Stocks were diluted in medium to a final concentration of 5 µM/ml. Inhibitor SiS3 (Merck Millipore) was dissolved 15 mg/ml in DMSO and stored at −20°C. Stocks were diluted in medium to a final concentration of 2 µM/ml. Experiments with vehicle DMSO were conducted with adjusted concentrations.

### Tracer diffusion assay

After 24 h of TGFβ treatment, fresh medium supplemented with TGFβ and 5 mg/ml FITC-coupled Dextran, MW 4 (FD4, Sigma-Aldrich) was loaded into the upper compartment of the insert. FD4 has a radius of 14 Å and, thus, can pass a cell monolayer only paracellularly. Medium change with TGFβ complementation was also applied in the lower compartment. After another 24 h of incubation and careful handling, a 100 µl sample was taken from the lower compartment and measured in a dilution series. Fluorescence intensity at wavelength extinction 490 nm/emission 520 nm was measured by ELISA reader (Tecan, Männedorf, Switzerland) combined with a black 96-well chimney plate (Greiner). Permeability coefficient (*P_app_*) was calculated using following equation: *P_app_ = (1/A·C_0_)·(dQ/dt)* with *dQ/dt* as the solute flux (*dQ* amount of relocated tracer and *dt* time span), *A* as the surface of the insert membrane and *C_0_* as the initial concentration.

### Immunofluorescence

Cells were fixed in 4% saline buffered paraformaldehyde for 10 min and blocked with 2% NGS for one hour. Polyclonal antibodies diluted in saline buffered 0.2% BSA/0.1% NaN_3_ against claudin-1 (Invitrogen, Camarillo, CA, USA, dilution 1∶150) were applied overnight at 4°C. Slides were washed and incubated with secondary antibody (Alexa goat-anti-rabbit Fluor 488, Invitrogen, Camarillo, CA, USA, dilution 1∶500) and DAPI (Roche, Indianapolis, IN, USA, 1∶2000) for 2 h at room temperature. After washing and glycerol imbedding, photos were made using Axioskop 2 plus (Carl Zeiss, Göttingen, Germany) supplied with AxioCam MRc (Carl Zeiss) and AxioVision, version 4.8 (Carl Zeiss).

### Generation of cell lysates

Cells (3×10^5^ cells/cm^2^) were seeded on ThinCert inserts (Greiner) with 0.4 µm pore size in 6-well plates (each insert 4.254 cm^2^). After 24 h of starvation with 2% FCS, TGFβ-1, -2 or -3 (Promokine) were added in a final concentration of 10 ng/ml combined with fresh medium. After 24 h in the presence or absence of TGFβs, filters were rinsed once with ice-cold PBS. The cells were scraped off with a flexible cell scratcher (TPP, Trasadingen, Switzerland) into 1 ml of ice-cold PBS, transferred into tubes and centrifuged for 10 min at 5000 g. Supernatant fractions were removed, and cell pellets were dissolved in 150 µl lysis buffer (20 mM Tris-Cl, pH 7.5, 150 mM NaCl, 1 mM EDTA, 1 mM EGTA, 1% Triton X-100), containing 7 µl/ml protease inhibitor cocktail (P1860, Sigma). Samples were kept on ice for 30 min and frequently vortexed and centrifugation at 10 000 g for 10 min at 4°C in order to remove cell debris and nuclei. The supernatant fractions, representing the cell lysates, were stored at −80°C until usage for Western blot analyses.

Membrane and cytosolic protein fractions from mouse epididymis were prepared as described previously [31]. After homogenization by ten strokes in a Potter–Elvehjem homogenizer (Wheaton, Millville, NJ, USA), samples were centrifuged at 3000 g for 8 min at 4°C to remove cell debris and nuclei. The supernatant fractions were ultra-centrifuged for 30 min at 100 000 g at 4°C. The resulting supernatant represents the cytosolic fraction. The pellets, representing the membrane fraction, were resuspended in 50 mM Tris-HCl buffer, pH 7.5, and stored at −80°C.

In all samples, protein concentrations were determined by using a kit from Bio-Rad (Munich, Germany) with bovine serum albumin (Sigma) as standard.

### Immunoblotting

After separation by SDS-PAGE, proteins were transferred to nitrocellulose membranes (Amersham, Braunschweig, Germany) at 30 V for 12–14 h at 4°C. Blots were stained with Ponceau S (P7170, Sigma), and the positions of co-migrated reference proteins (SDS-6H, Sigma) were marked. Records of the protein images served as additional protein loading control. The blots were then treated for 2 h with blocking solution (Roche, Mannheim, Germany) prior to probing with antibodies directed against claudin-1 (Invitrogen, 1∶2000), occludin (Invitrogen, 1∶2000), and vinculin (Sigma, 1∶6000). Either anti-mouse or anti-rabbit IgG, linked to peroxidase (Pierce, Rockford, IL, USA), were used as secondary antibodies. Signals were detected using enhanced chemiluminescence (Amersham, RPN 2105) on Fuji (13862 C) X-ray films. After stripping of bound antibodies [32], blots were re-used for detection of other proteins, especially vinculin, which was used for the normalization of densitometric measurements.

### Statistics

All data were gained from three independent experiments conducted in duplicate. Mean values and SEM were calculated using MS Excel, Version 2010. Calculation of p-values was done by Students t-test using Excel or non-parametric Mann-Whitney-test using GraphPad Prism 5 (GraphPad Software, La Jolla, CA, USA).

## Results

### TGFβs mediate disruption of epididymal barrier *in vitro*

In order to investigate the influence of TGFβs on the barrier of the epididymal epithelium, confluent monolayers of MEPC5 cultured on inserts were treated after two days of culture with one of the three TGFβ isoforms, which were added in both, the apical and the basolateral compartment of the transwell inserts.

**Figure 1:**
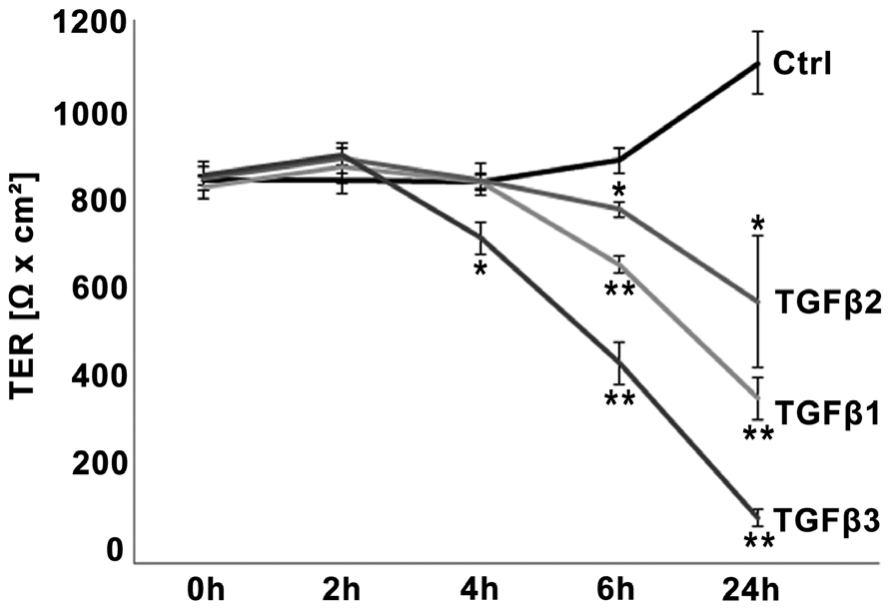
Time-dependent TGFβ effects on TER in the BEB *in vitro* model. TGFβs decreased epithelial barrier in a time-dependent manner. TGFβ3 decreased absolute values of TER (indicated by Ω x cm^2^) significantly within 4 h, 6 h and 24 h compared to untreated control each. TGFβ1 and TGFβ2 influenced the TER significantly after 6 h and 24 h. Data points represent mean values obtained from n = 6, generated by three independent repetitions performed in duplicate. SEM is indicated, p-values ≤0.05 (Mann-Whitney-test) were considered significant (_*_), p≤0.005 highly significant (_**_).

The TER value at day 2 (0 h/initial value) was on average of 837 Ω x cm^2^±11.2 (Fig. 1). After 24 h (day 3) the TER increased to 1092 Ω x cm^2^±68.7 in the untreated control (Fig. 1A). All three isoforms of TGFβ decreased the TER. Compared to control, treatment with TGFβ3 significantly decreased the TER to 710 Ω x cm^2^±34.1 after 4 h, 437 Ω x cm^2^±47.4 after 6 h and 98 Ω x cm^2^±18.7 after 24 h, respectively (Fig. 1A). Treatment with TGFβ1 and TFGβ2 decreased TER significantly within 6 h (TGFβ1: 652 Ω x cm^2^±18.8; TGFβ2: 773 Ω x cm^2^±15.8) and 24 h (TGFβ1: 360 Ω x cm^2^±45.9; TGFβ2: 571 Ω x cm^2^±145.5). Regularly, TGFβ2 effects were less pronounced than TGFβ3 and TGFβ1 effects, but have to be considered as significant after 24 h compared to control due to increase of TER in the control. When TGFβs were removed after 24 h of incubation the effect could be partially reversed (data not shown).

Since the initial TER values (0 h) regularly showed slight variations, even between duplicates, in all following experiments the TER values were normalized to the initial value (0 h) of each individual insert (100%). This normalization did not change the level of significance compared to calculation by absolute values (data not shown).

**Figure 2:**
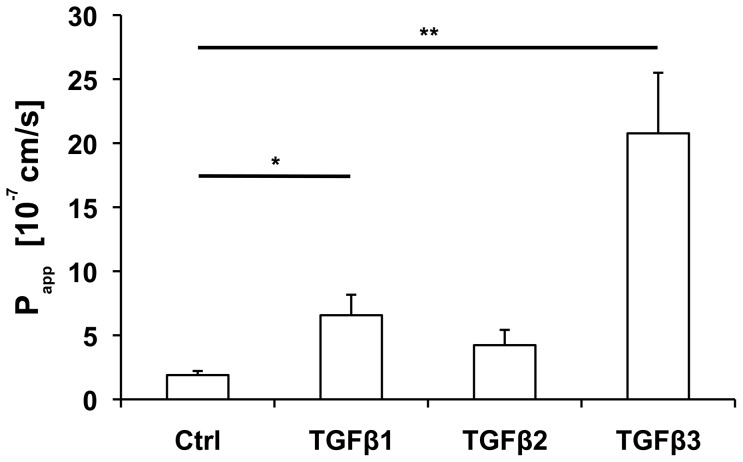
TGFβ effects on tracer diffusion in the BEB *in vitro* model. Treatment with TGFβ3 increased the permeability coefficient (P_app_) highly significant (p = 0.0002) compared to untreated control, whereas for TGFβ1 the level of significance (p = 0.0281) is lower. Each column represents a mean value obtained from n = 6, generated by three independent repetitions performed in duplicate. SEM is indicated, p-values ≤0.05 (Mann-Whitney-test) were considered significant (_*_), p±0.005 highly significant (_**_).

In the tracer diffusion assay, monolayers were pretreated for 24 h with TGFβs before TGFβs together with FD4 were applied for another 24 h. The amount of FD4 diffusing through the monolayer (measured by the permeability coefficient, *P_app_*) treated with TGFβ3 was about 10fold higher (highly significant, p = 0.0002) compared to an untreated monolayer (Fig. 2). TGFβ1 also showed impressive effects on the capacity of diffusion, whereas TGFβ2 effects were negligible (TGFβ1, p = 0.0281; TGFβ2, p = 0.2072). These results mirror the findings from TER measurements, indicating that the TER actually reflects the paracellular permeability of this *in vitro* model.

### TGFβs effects on cell numbers, viability and morphology

**Figure 3:**
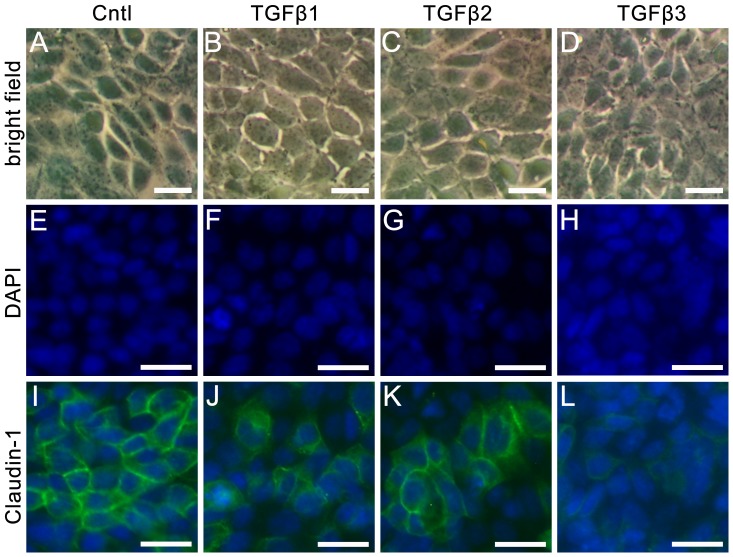
Cell morphology and claudin-1 immunostaining after treatment with TGFβs. MEPC5 monolayers photographed in bright field (A–D), DAPI staining (E–H), and claudin-1 immunostaining (I–L). A: untreated MEPC5; B–D: MEPC5 cells treated with TGFβs for 24 h. No obvious changes in cell morphology are detectable. E: DAPI labeling of the nucleus in untreated MEPC5; F–H: MEPC5 cells treated with TGFβs for 24 h. No obvious changes are detectable. I: Immunostaining of claudin-1 in untreated MEPC5, DAPI labeling of the nucleus. The pattern of claudin-1 localization shows numerous cell clusters characterized by an intense immunofluorescence at the cell borders. J–L: MEPC5 cells treated with TGFβs for 24 h. After treatment with TGFβ3 (and to a lesser intent with TGFβ1) the staining was generally weaker, the striking immunofluorescence at cell borders was clearly reduced. Photos are examples from at least three independent experiments. Scale bar: 12.5 µm in A–D, 25 µm in E–L.

To exclude that changes of paracellular permeability by TGFβs are the consequence of TGFβ-dependent cell number variation, cells were counted using BioRad Cell Counter System. Compared to controls, treatment for 24 h with TGFβ1, TGFβ2 and TGFβ3, respectively, showed only slight effects on cell numbers, which were not significant (all p-values >0.15, Fig. S1). Combination of cell counting with Trypan blue staining did not give any hint for increased cell death after TGFβ treatment (data not shown). In addition, the phenotypic shape of the cells did not change under culture conditions with TGFβs (Fig. 3A–D). According to these results, TGFβs do not affect cell numbers, cell viability or morphology to an extent that is relevant for permeability.

### Changes of localization and protein expression of tight junction proteins after TGFβ treatment

Following the axiom, that the paracellular barrier is mediated by tight junctions, the localization of tight junction proteins that were described to be expressed in the epididymis were checked on TGFβ-treated monolayers of MEPC5 cells (Fig. 3). In untreated controls (Fig. 3I) the pattern of claudin-1 localization showed numerous cell clusters comprising six or more cells, characterized by an intense immunofluorescence at the cell borders. This pattern is comparable to the well known staining of tight junctions in tangentially cut epithelial cells from tissues sections. We found such a staining pattern for claudin-1 also in murine epididymis sections (data not shown).

After the application of TGFβs, this pattern is disturbed (Fig. 3K–L). Especially treatment with TGFβ3 (Fig. 3L) resulted in essential changes of the claudin-1 staining pattern. Larger cell clusters and the striking immunofluorescence of cell borders were barely detectable. These TGFβ-dependent changes are not due to reduced cell numbers as shown by (i) cell counting (see above, Fig. S1), (ii) bright field photographs (Fig. 3A–D) and (iii) additional DAPI stainings (Fig. 3E–H).

**Figure 4:**
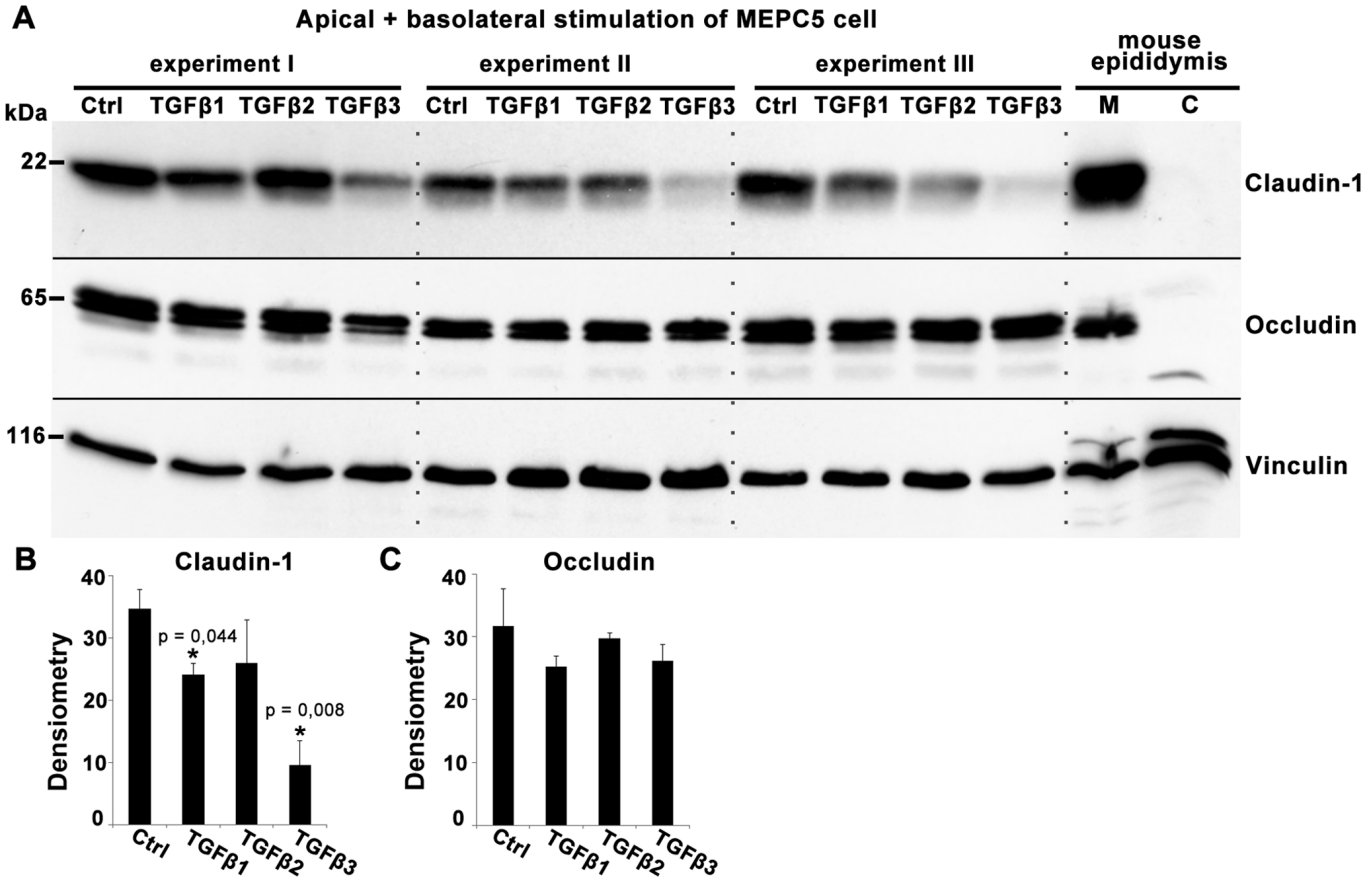
Western blot analysis of claudin-1 and occludin expression after treatment with TGFβs. A: Western blot with lysates of MEPC5 after combined basolateral and apical stimulation with TGFβ1, TGFβ2 or TGFβ3 for 24 h compared to untreated cells. Lysates from three independent experiments (I–III) were used. TGFβ1 and especially TGFβ3 decreased the level of claudin-1 compared to the untreated sample. The levels of occludin were unaffected. Membrane (M) and cytosolic (C) protein fractions of mouse epididymis served as controls. B, C: Densitometric analysis of claudin-1 (B) and occludin (C) protein expression. Claudin-1 showed a significant reduction of expression by TGFβ3 by TGFβ1 treatment (p±0.05). All arbitrary units were normalized to the corresponding values of vinculin, serving as loading control. Columns represent mean values of three independent experiments (I–III) with SEM indicated. p-values compared to untreated controls according to Students t-test.

Western blot analyses of lysates from MEPC5 cells treated in 6-well inserts from the apical and basolateral side (Fig. 4A) revealed a significantly reduced expression of claudin-1 after treatment with TGFβ1 and TGFβ3 (Fig. 4B), whereas the expression of the tight junction protein occludin was unchanged (Fig. 4C). Control experiments using mouse tissue confirmed expression of claudin-1 and occludin in the mouse epididymis and showed their predominant localization in the membrane fraction (Fig. 4A, right lanes). Vinculin served as standard for normalization of claudin-1 and occludin expression in densitometric measurements (Fig. 4B, 4C).

### TGFβ-receptor-1 kinase and Smad3 are involved in TGFβ-dependent changes of TER

**Figure 5:**
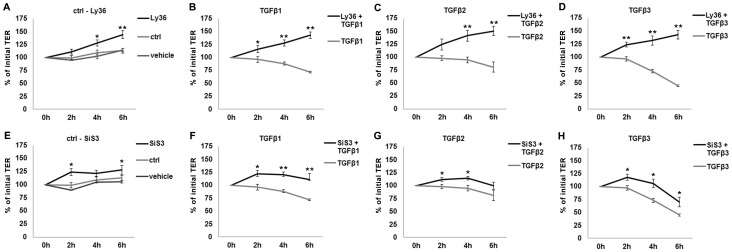
Time-dependent effects of TGFβ pathway inhibitors on TER in the *in vitro* model. In case of inhibitor application, cells were incubated with inhibitors alone for 3βs + inhibitors for 2 h, 4 h and 6 h. Values prior to any treatment were taken as baseline (100%). A: The inhibition of TGFβ-R1 kinase activity by Ly364947 (Ly36) compared to untreated and vehicle-treated cells. B-D: TGFβs-affected TER values in the presence and absence of Ly364947. Effects observed for TGFβs are significantly inhibited by TGFβ-R1 inhibition. E: The inhibition of Smad3 by SiS3 compared to untreated and vehicle-treated cells. F–H: TGFβs-affected TER values in the presence and absence of SiS3. Inhibition of Smad3 resulted in attenuation of TGFβ effects. Data points represent mean values obtained from n = 6, generated by three independent repetitions performed in duplicate. SEM is indicated, p-values ±0.05 (Mann-Whitney-test) were considered significant (_*_), p±0.005 highly significant (_**_).

To make sure that the effects observed are TGFβ-R1-dependent, Ly364947, an inhibitor of TGFβ-R1 kinase, was applied 3 h prior to TGFβ stimulation. The inhibition of TGFβ-R1 kinase was able to fully prevent the effects of all three TGFβs (Fig. 5A–D), indicating that all effects of TGFβs are mediated by TGFβ-R1 kinase activity.

In the next step, we analyzed how the effects are mediated downstream of the TGFβ receptor complex. Inhibition of Smad3 phosphorylation by the inhibitor SiS3 attenuated the effects of the TGFβ isoforms (Fig. 5E–H), indicating that Smad3 is involved in increased TGFβ-mediated permeability. Inhibition of non-canonical TGFβ pathways via JNK did not have any significant effect on TER (data not shown).

### Epididymal epithelial cells are responsive to TGFβ predominantly from basolateral side

**Figure 6:**
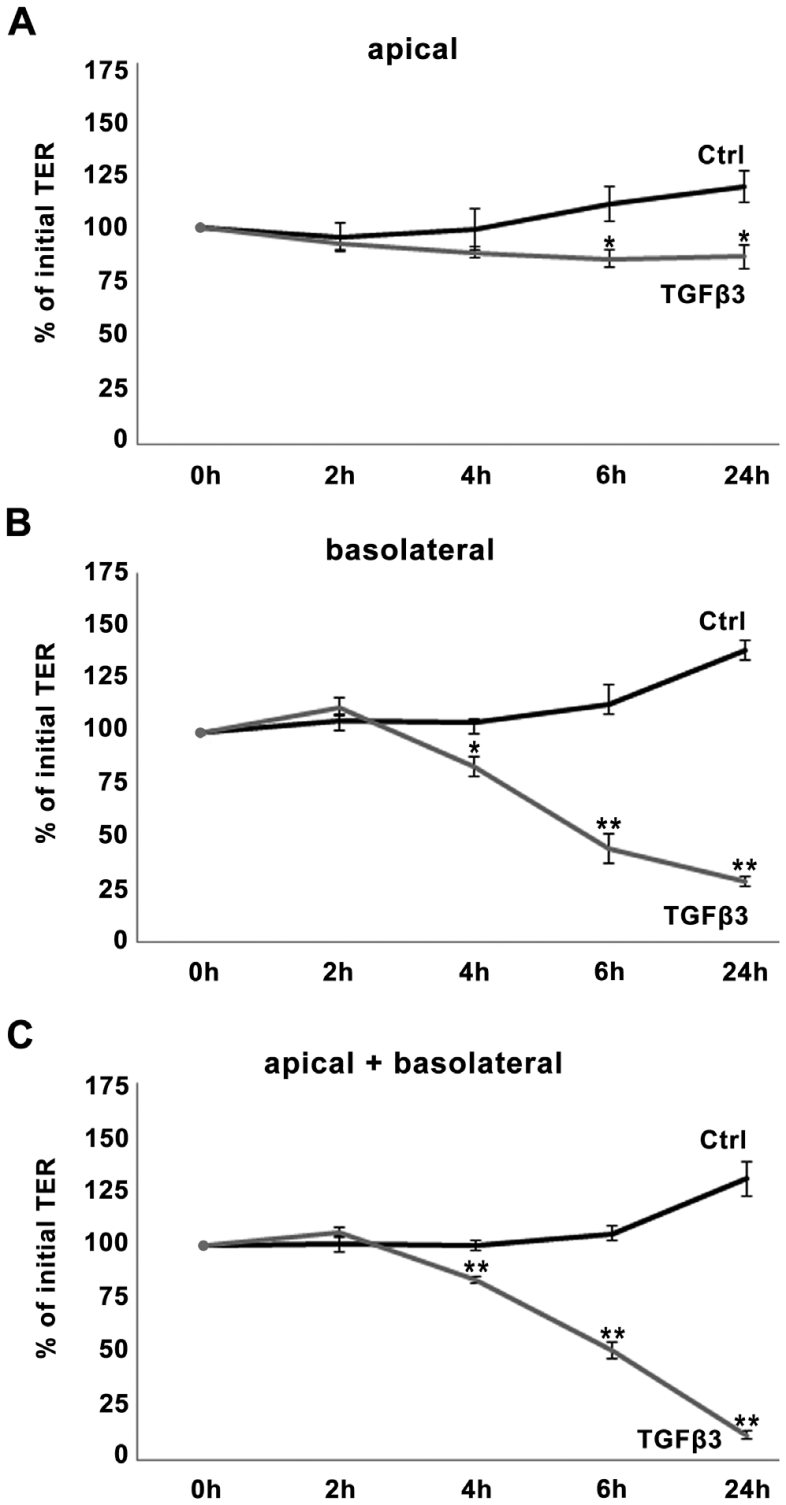
Dependency of TGFβ effects on TER from the side of stimulation. TGFβ3 was applied into the upper compartment (apical) only (A), into the lower compartment (basolateral) only (B), or into both (apical + basolateral), (C). Basolateral application of TGFβ3, whether alone or together with apical stimulation decreased TER significantly at 4 h, 6 h and 24 h compared to untreated control, whereas apical stimulation had only slight effects. Due to increase of TER in the untreated control the effect of apical stimulation has to be considered significant after 6 h and 24 h. Data points represent mean values obtained from n = 6, generated by three independent repetitions performed in duplicate. Value prior to treatment was taken as baseline (100%). SEM is indicated, p-values ±0.05 (Mann-Whitney-test) were considered significant (_*_), p±0.005 highly significant (_**_).

Since MEPC5 cells are strongly polarized [29], we used the MEPC5 model to analyze whether the epididymal epithelium is predominantly responsive to the TGFβ signals from luminal (apical) or stromal (basolateral) side. For this, TGFβ was added either to the apical, or to the basolateral or to both compartments (Fig. 6).

Stimulation from basolateral side with TGFβ3 resulted in effects similar to those observed for simultaneous stimulation from both sides, decreasing the TER significantly at the data points 4 h, 6 h and 24 h (Fig. 6B and 6C). Effects after apical stimulation (Fig. 6A) were barely detectable. Even after 24 h the TER remained in the range of the initial value. Only due to a slight increase of TER in the untreated control, TER effects became significant after 6 h and 24 h.

According to these data, MEPC5 cells are predominantly responsive to TGFβ3 from the basolateral side, suggesting that TGFβ3 might affect the permeability of the epididymal epithelium *in vivo* predominantly from the interstitial side. Similar but less pronounced results were obtained for TGFβ1 (data not shown).

### Protein expression of the tight junction proteins claudin-1 and occludin after TGFβ treatment from different sides

**Figure 7:**
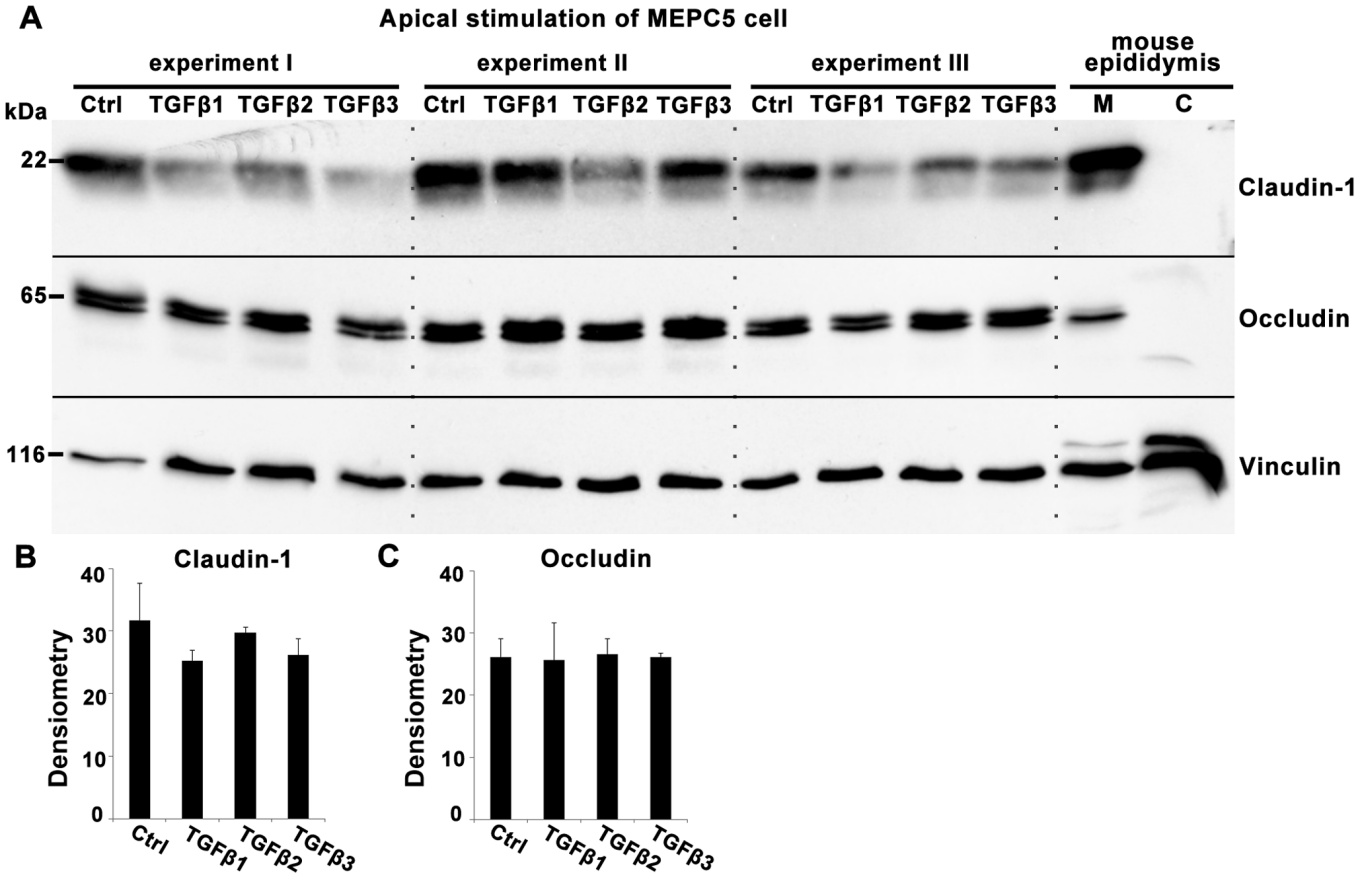
Western blot analysis of claudin-1 and occludin expression after apical treatment with TGFβs. A: Western blot with lysates of MEPC5 after apical stimulation with TGFβ1, TGFβ2 or TGFβ3 compared to untreated cells. TGFβ1 and TGFβ3 decreased the level of claudin-1 slightly compared to the untreated sample. The levels of occludin were unaffected. Membrane (M) and cytosolic (C) protein fractions of mouse epididymis served as controls. B, C: Densitometric analysis of claudin-1 (B) and occludin (C) protein expression. Claudin-1 showed a modest reduction of expression by TGFβ1 and TGFβ3 treatment which is not significant (p>0.05). All arbitrary units were normalized to the corresponding values of vinculin, serving as loading control. Columns represent mean values of three independent experiments with SEM indicated. p-values compared to untreated controls according to Students t-test.

In order to test if the distinct side-specific TGFβ effects on TER correspond to tight junction protein levels, the expression of claudin-1 was quantified by Western blot. Lysates were gained from cells cultured on 6-well inserts and identical amounts of proteins were analyzed by Western blot (Fig. 7). Whereas combined stimulation from the basolateral and apical side resulted in a strong decrease of claudin-1 levels by TGFβ3 and TGFβ1 (see Fig. 4A, 4B), stimulation from the apical side alone showed only slight changes of claudin-1 expression (Fig. 7A, 7B), which were not significant (p>0.05). The level of occludin was not affected by apical (Fig. 7A, 7C) or apical/basolateral treatments (see Fig. 4A, 4C). Vinculin served as standard for normalization of claudin-1 and occludin expression in densitometric measurements (Fig. 7B, 7C).

## Discussion

### Our *in vitro* system – TER and Tracer diffusion as benchmarks of paracellular permeability

Based on our preparatory experiments [29] we established a BEB *in vitro* model. In this model a highly efficient barrier of 840 Ω x cm^2^ was produced, which is in the range of TER observed in gut epithelium *in vitro* models [33] and is higher than described for *in vitro* models of the blood-testis barrier with less than 250 Ω x cm^2^ [34], [35]. In *in vitro* models of renal proximal tube up to 240 Ω x cm^2^ were observed [36].

Furthermore, we were able to modulate the BEB *in vitro* in a highly reproducible manner, thus we established a robust system that allows screening of substances affecting the BEB in future studies. Our permeability experiments were based on two entities, electrical resistance and passive diffusion. The measurement of the electrical resistance is a widely accepted method to evaluate epithelial barriers, but TER cannot discriminate between paracellular and transcellular ion flux [12]. Evaluation of tracer diffusion measures paracellular permeability exclusively, because FD4 has a radius of 14 Å and can pass through the monolayer only paracellularly [12]. In our experiments, both methods resulted in comparable conclusions about the distinct effects of the three TGFβ isoforms, indicating a high reliability.

### Different TGFβ isoforms have a distinct influence on the BEB

For the first time, we demonstrate that the permeability of the BEB can be affected by cytokines of the TGFβ family. We found that the permeability of the BEB can be influenced by the TGFβ isoforms differentially. An influence of TGFβs on epithelial paracellular permeability has been shown for other organs, whereas a screening for the differential effect of the distinct isoforms has not been conducted in any other organ before.

Our observations suggest that the three TGFβ isoforms differ in their effect on paracellular permeability of the BEB. The strongest effect within a few hours was found for TGFβ3. TGFβ3 has also been reported to have a strong effect on the blood-testis barrier [35]. TGFβ1 affected the BEB less pronounced. However, the effect of TGFβ2 was rather weak. These findings correspond to the differing TGFβ-R2 affinity of the three isoforms, with TGFβ3 having the highest affinity, the affinity of TGFβ1 being less strong and the affinity of TGFβ2 being rather weak [37]. All effects on permeability were demonstrated to be TGFβ-R1-dependent, which activates the Smad pathway after dimerization with TGFβ-R2 after ligand interaction. The differing affinity of the TGFβ isoforms to TGFβ-R2 might be one of the reasons for the gradual differences of effects.

### Regulation of paracellular permeability by tight junctions

The tightness of epithelia is a balance between forming of compartments (“barrier”) and paracellular permeability in order to fulfill specific functions (“fence”). Paracellular flux is regulated by tight junction proteins on the cell surface. The mechanism of regulation of tight junction quantity is discussed controversially. Most authors suppose a regulation mediated by degradation or endocytosis of tight junction proteins [14]. However, transcriptional control of tight junction biosynthesis was also suggested to be involved in the regulation of barrier tightness [38].

The immunofluorescence and Western blot revealed that the level of claudin-1 protein is reduced after treatment with TGFβ1 and especially TGFβ3, mirroring the ratios found in the permeability assessment. In contrast to claudin-1, occludin was not affected significantly, suggesting that the tight junction proteins claudin-1 and occludin are differently influenced by TGFβs. Claudin-1 reduction is associated with the breakdown of the barrier. Occludin cannot rescue this effect.

Of note, claudin-1 knockouts die perinatally because the impaired epithelial barrier results in dehydration [6]. In contrast, occludin knockout mice are viable and show intact epithelial barriers in the intestine and bladder [8], but occludin knockout male mice are infertile [9]. Our data do not provide any evidence that the infertility of occludin knockout mice is due to dysfunction of the epididymal epithelium.

### Side-specific effects of TGFβs

In our experiments, we found that the cultured epididymal epithelial cells are predominantly responsive to TGFβ3 from the basolateral side. Stimulation from the apical side did not show significant effects neither on TER nor on claudin-1 expression.

Transferring these data into the *in vivo* situation, the basolateral stimulation would represent TGFβ signal from interstitial tissue. The stroma of the epididymis comprises reservoirs of growth factors such as TGFβs in the ECM of the connective tissue [26], [27]. TGFβ3 was described to be predominantly present in corpus region [26] and has the strongest effect of the three isoforms on paracellular permeability. TGFβ1, which was found in all parts of the epididymal stroma [26], has a strong effect on paracellular permeability. However, TGFβ2, which seems not to be present in the epididymis [26], had the slightest effect on the permeability of the epididymal epithelium compared to the other two isoforms. Interestingly, Desai and coauthors [26] did not detect the active form of TGFβ1 in the epididymis, suggesting activation of stored TGFβ1 might be restricted to distinct situations, such as infection or inflammation of the epididymis. *In vivo*, the epididymal epithelium could be affected by TGFβ activation from the stroma. In contrast, TGFβs, produced in the testis, and presumably present in the luminal fluid of the epididymis, might not have relevant effects on the epididymal epithelium.

The function of the high amounts of inactive TGFβ in the stroma has not been elucidated so far. We speculate that activation of TGFβs might provide a mechanism to loosen the BEB in order to allow migration of dendritic cells into the epididymal epithelium. The presence of dendritic cells in the epididymal epithelium has been described, whereas the mechanisms of migration remained unresolved [39].

In conclusion, our experiments show that TGFβs modulate the permeability of the BEB. Comparing the three isoforms, TGFβ3 has an outstanding strong effect on the permeability which corresponds to strong reduction of claudin-1 levels. Inhibitor experiments suggest a TGFβ-R1 kinase- and Smad-dependent mechanism. Our data provide a mechanism how the tightness of the blood-epididymis barrier is modulated. We developed a robust BEB *in vitro* model that can path the route of investigation to the role of BEB in male fertility.

## Supporting Information

Figure S1 **Influence of TGFβs on the number of MEPC5 cells.** No significant difference was observed comparing the cell number after 24 h treatment with TGFβs and control evaluated by automated cell counting. Data points represent mean values obtained from n = 6, generated by three independent repetitions performed in duplicate. SEM is indicated, p-values ≤0.05 (Mann-Whitney-test) were considered significant (_*_), p≤0.005 highly significant (_**_).
(TIF)
