# Supplementary figures and images for: TGFβs Modulate Permeability of the Blood-Epididymis Barrier in an In Vitro Model
Source: PLoS One. 2013 Nov 13;8(11):e80611. doi: 10.1371/journal.pone.0080611 (PMC3827453; doi:10.1371/journal.pone.0080611)

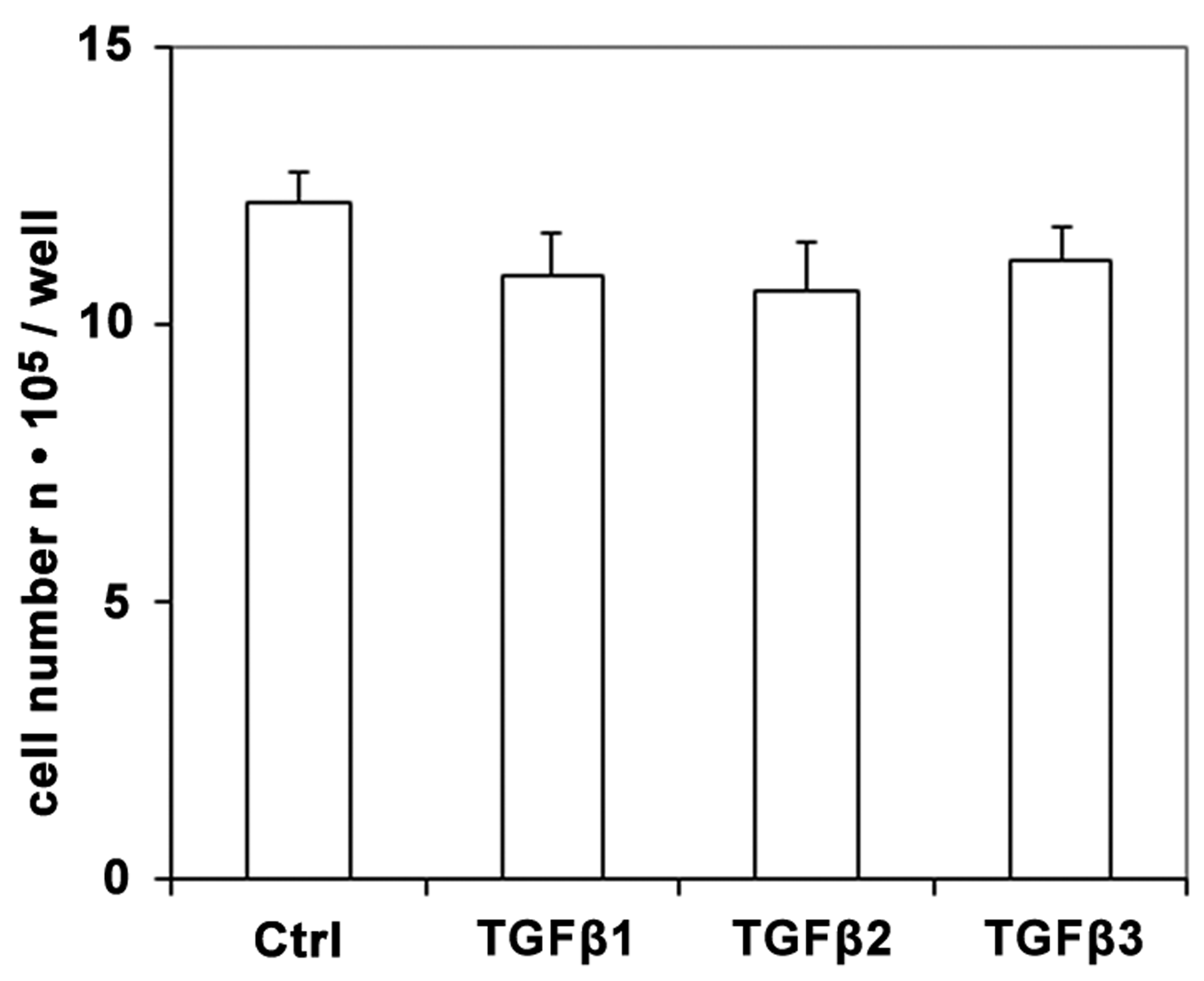

Supplement: Figure S1 — Influence of TGFβs on the number of MEPC5 cells. No significant difference was observed comparing the cell number after 24 h treatment with TGFβs and control evaluated by automated cell counting. Data points represent mean values obtained from n = 6, generated by three independent repetitions performed in duplicate. SEM is indicated, p-values ≤0.05 (Mann-Whitney-test) were considered significant (*), p≤0.005 highly significant (**). (TIF) [file pone.0080611.s001.tif]
